# MDAPT: Multi-Modal Depth Adversarial Prompt Tuning to Enhance the Adversarial Robustness of Visual Language Models

**DOI:** 10.3390/s25010258

**Published:** 2025-01-05

**Authors:** Chao Li, Yonghao Liao, Caichang Ding, Zhiwei Ye

**Affiliations:** 1 School of Computer Science, Hubei University of Technology, Wuhan 430068, China; lichao@hbut.edu.cn (C.L.); 102301175@hbut.edu.cn (Y.L.); hgcsyzw@hbut.edu.cn (Z.Y.); 2 School of Computer and Information Science, Hubei Engineering University, Xiaogan 432000, China

**Keywords:** multi-modal, adversarial robustness, visual language models, prompt tuning

## Abstract

Large visual language models like Contrastive Language-Image Pre-training (CLIP), despite their excellent performance, are highly vulnerable to the influence of adversarial examples. This work investigates the accuracy and robustness of visual language models (VLMs) from a novel multi-modal perspective. We propose a multi-modal fine-tuning method called Multi-modal Depth Adversarial Prompt Tuning (MDAPT), which guides the generation of visual prompts through text prompts to improve the accuracy and performance of visual language models. We conducted extensive experiments and significantly improved performance on three datasets (ϵ=4/255). Compared with traditional manual design prompts, the accuracy and robustness increased by an average of 17.84% and 10.85%, respectively. Not only that, our method still has a very good performance improvement under different attack methods. With our efficient settings, compared with traditional manual prompts, our average accuracy and robustness are improved by 32.16% and 21.00%, respectively, under three different attacks.

## 1. Introduction

With the rapid development of large model research, large pre-trained Visual Language Models (VLMs) such as CLIP [1], ALIGN [2], BLIP [3],ViLT [4], etc., have shown excellent generalization ability to downstream tasks. These models are designed to align language and vision modalities using web-scale datasets, such as the 400 million text–image pairs utilized in CLIP. As more and more research work builds on these models, the vulnerabilities inherent in these models can have serious consequences. One critical issue revealed by the recent studies [5,6,7,8] is that these VLMs, like vision models, are highly susceptible to adversarial attacks. Therefore, the model robustness of adversarial attacks is now a major focus of research.

Initial pre-training of large VLMs typically needs to be performed on large datasets and then adapted to specific downstream tasks. Adapting to the downstream task is very important and determines what the main task of the model is and how efficiently the downstream task can be handled. There are many ways to fit downstream tasks, fine-tuning model weights [9] being the most common. However, as the parameters of pre-trained models become larger and larger, especially for networks based on ViT-Huge [10] (632 M parameters), ResNet-50 [11] (25 M parameters), etc., fully fine-tuning the pre-trained model is an expensive and infeasible approach and may be even more unaffordable if adversarial training [12] is applied to improve adversarial robustness. Not only that, fine-tuning the pre-trained model may also impair the generalization performance of the model. Therefore, freezing all or most of the parameters of the pre-trained model, such as CoOp [13], becomes a very promising approach.

**Figure 1:**
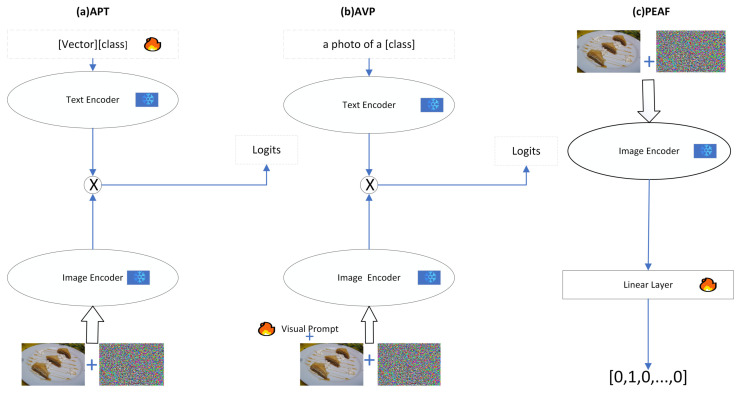
The frost represents the freezing parameter and the flame represents the updatable parameter: Figure 2 is our model, compared with figure (**a**), which is APT, Figure (**b**), which is AVP, and Figure (**c**), which is Partial Adversarial Fine-Tuning (PEAF) [14].

Current adaptive approaches to adversarial robustness focus on model weights, adversarial fine-tuning [14,15,16], or image pixels. The main approach for image pixels is Adversarial Visual Prompting (AVP) [17,18]. Inspired by Natural Language Processing (NLP), the literature (Adversarial Prompt Tuning, APT) [19] proposes a textual prompt tuning approach by investigating the impact of the adversarial robustness of textual prompts. Figure 1 shows the above mentioned current main modeling approaches. It is worth mentioning that although adversarial attacks have a significant impact on VLMs, previous research has rarely focused on investigating their adversarial robustness.

**Figure 2:**
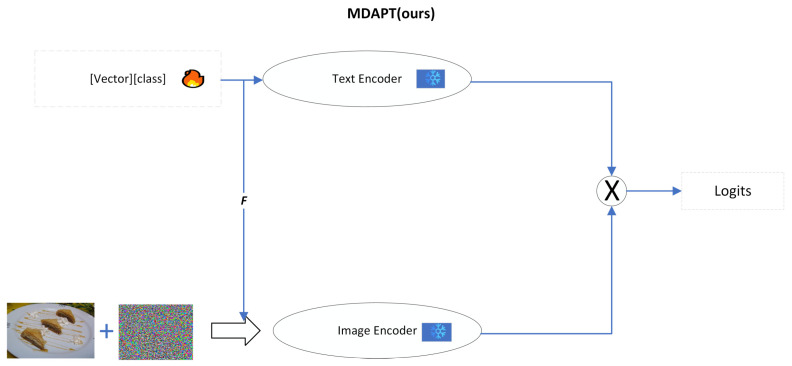
MDAPT advanced model architecture, text prompts, and visual prompts are learnable, and visual prompts are generated by mapping from text prompts via F functions.

Our motivation derives from the multi-modal nature of CLIP [1], where a text and image encoder co-exist and both contribute towards properly aligning the vision–language modalities. A single mode can not achieve better semantic alignment, and text prompts alone cannot improve the effectiveness of the adaptive requirements of image encoders. As far as the main working methods are concerned, APT [19] uses only textual prompts, AVP [17] uses visual prompts, and PEAF [14] discards language branching, only fine-tuning the final linear layer. Although existing methods have certain advantages, they have obvious limitations when dealing with adversarial prompt tuning in VLMs. This is because a single modality does not better tune the visual language features nor more effectively enhance image feature embedding in the clustering. Therefore, employing a multi-modal approach is crucial for enhancing the robustness of VLMs. Our work aims to improve the adversarial robustness of pre-trained models by investigating textual prompts for guided visual prompt generation through multi-modal ideas. In order to improve the adversarial robustness of pre-trained models, a new Multi-modal Depth Adversarial Prompt Tuning (MDAPT) method, as shown in Figure 2, is proposed. MDAPT achieves stronger semantic alignment by jointly training textual and visual prompts to ensure effective adversarial prompt tuning. This allows text and image features to complement each other, thus enhancing the robustness of the overall model. In this paper, we use CLIP [1], a typical visual language model, because it has been widely used and developed.

In summary, the main contributions of this work include the following:

- We propose a new Multi-modal Depth Adversarial Prompt Tuning (MDAPT) method to improve the robustness of large pre-trained visual language models.
- The MDAPT was evaluated through experiments on the Caltech101, Flowers102, and StanfordCars datasets.
- We demonstrates that our method is highly effective in improving the performance of pre-trained VLMs, achieving a good balance between accuracy and robustness.

## 2. Related Works

In this section, we first review in-depth research on VLMs, followed by a literature review on optimization methods and adversarial training methods. This will play a significant role in the development of our research. In addition, we emphasize the crucial role of multimodality in improving adversarial robustness.

### 2.1. Visual Language Models

Compared with the image supervised learning model, VLMs enrich the representation of multiple modes. More recently, large models of visual languages, such as CLIP [1], ALIGN [2], LIT [20] and FILIP [21], have shown excellent performance in a wide range of tasks, including few-shot and zero-shot image recognition.These models use a large amount of data from the network to learn the image text representation in a self-supervised learning method. These models are used for downstream tasks after being pre-trained. Reference [22] proposes a novel knowledge-guided semantic transfer network (KSTNet) for few-shot image recognition from a supplementary perspective by introducing auxiliary prior knowledge. Inspired by hierarchical human visual systems, reference [23] proposes the ConTriNet, a robust Confluent Triple-Flow Network employing a divide-and-conquer strategy. This framework utilizes a unified encoder with specialized decoders, each addressing different subtasks of exploring modality-specific and modality-complementary information for RGB-T SOD, thereby enhancing the final saliency map prediction.

### 2.2. Tune the Model to Fit the Precision Approach

In contrast to the traditional approach to fine-tune the entire model’s parameters [24], currently available methods for the efficient tuning of parameters fall into three main categories: prompt tuning [13], adapter tuning [25], and linear probing [26]. Text prompts are usually given to the VLMs of the language branch to make it better understand the task. Prompt designs can be made by hand for downstream tasks or they can be learned automatically during the fine-tuning phase. Prompt tuning is first used for NLP [27,28,29,30], then only visually [31,32,33,34], and finally for adaptation in VLMs [13,35]. Similar to the literature [31], our design also experimented with deep text and visual prompts to better improve the robustness of large pre-trained VLMs. Adapter tuning inserts a learnable micro-component into the model to adapt to downstream task training. Linear probing is achieved by training only the linear layers connected at the end of the model.

### 2.3. Adversarial Training

Adversarial training [36] is by far the most effective defense against adversarial examples [37]. It replaces clean examples with adversarial examples that are dynamically generated during training. But adversarial training is very expensive, and it is easy to over fit [38]. Therefore, people have proposed a large number of methods to solve the efficiency problem, such as references [39,40,41,42]. However, most of the current work concerns how to train the model from the beginning, which makes the efficiency of adversarial training tuning low. Therefore, it is very important to study the use of pre-trained models for adversarial robustness, but relevant work is not common at present. Most of the current work adjusts the robustness of pre-trained models through adversarial fine-tuning. For example, reference [43] proposes a new method for efficiently detecting machine-generated text by applying adversarial training to pre-trained language models, and reference [44] proposes a new statistics-based approach for improving adversarial robustness: the Two-channel Normalization (TWINS) fine-tuning framework. Model weights are fine-tuned through adversarial training. Depending on the number of parameters that need to be adjusted, these methods are divided into full adversarial fine-tuning and partial adversarial fine-tuning [14]. Reference [17] adjusts the adversarial robustness of the pre-trained model by using adversarial visual prompts, and reference [19] adjusts the adversarial robustness of the pre-trained model by studying text prompting. Reference [45] proposes a contrast-adversarial training method to increase visual involvement in semantic representation learning, and by comparing multi-modal inputs with adversarial samples, the model learns to recognize the samples with the most useful information. Reference [46] proposes the Transferable Multi-Modal (TMM) attack framework, which tailors both the modality consistency and modality discrepancy features. Our approach is designed to adversarially adjust the pre-trained model by adjusting the multi-modality of the text prompting to generate a visual prompting, unlike the adversarial full fine-tuning, partial fine-tuning, or adversarial visual prompting work described above.

## 3. Method

In this section, we first define our method: Multi-modal Depth Adversarial Prompt Tuning (MDAPT). Afterwards, we provide a detailed explanation of the core components: CLIP model, multi-modal construction method, and adversarial training optimization method.

### 3.1. Review of CLIP

**Figure 3:**
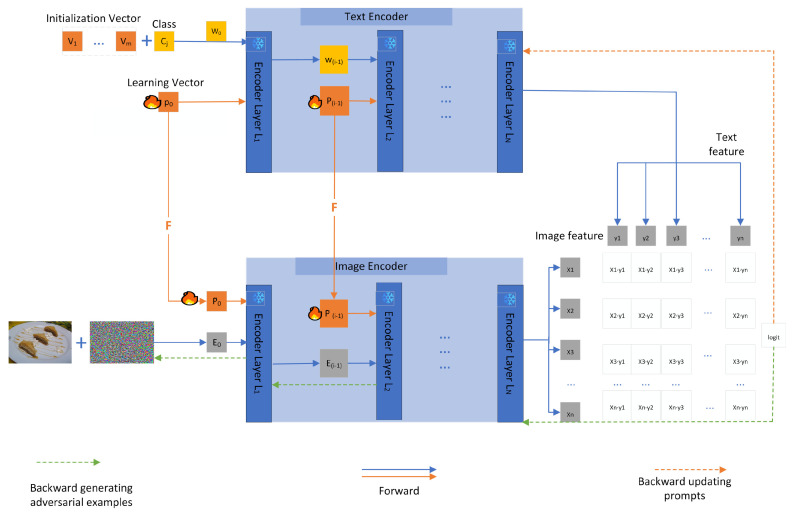
The framework of the MDAPT model proposed by ourselves using a visual language model is summarized. Both image and text are frozen; only prompt text and visual prompt is learnable. MDAPT maps text prompts to visual prompts using the *F* function. We use deep text and vision prompts to learn across multiple transfomer modules. At the same time, adversarial samples are generated backwards.

Our approach is built on a pre-trained VLM, CLIP [1], as shown in Figure 3, which consists of two primary components: an image encoder and a text encoder, parameterized by $\theta_{v}$ and $\theta_{t}$, respectively. CLIP encodes the image $I\in R^{H\times W\times 3}$ and the corresponding text description. These are used to extract the features from images and text, respectively. Given an input image $x_{i}$ and text $t_{j}$, the respective features $z_{v}^{i}$ and $z_{t}^{j}$ are computed as described below:

(1) $$z_{v}^{i}=f\left(x_{i};\theta_{v}\right),\;z_{t}^{j}=f\left(t_{j};\theta_{t}\right)$$

Cosine similarity scores are then calculated for each pair of image and text features to measure how well they align:

(2) $$s_{i,j}=\cos\left(z_{v}^{i},z_{t}^{j}\right)$$

Calculating these similarity scores is similar to the logit output of classical visual models such as VIT [10]. The probability that $X_{i}$ is aligned with $t_{j}$ is:

(3) $$p_{i,j}=p\left(x_{i},t_{j}\right)=\frac{\exp\left(s_{i,j}\right)}{\sum_{j}\exp\left(s_{i,j}\right)}$$

The two encoders jointly pre-train by maximizing the similarity score of the real image–text pair, i.e., *i* = *j*, while minimizing the similarity score of the error pair. Once pre-trained, CLIP can be applied to perform zero-shot image classification by using the text description of the classes in the target dataset as a text prompt and predicting the most likely classes:

(4) $$\arg\max_{j}p_{i,j}$$

By default, CLIP uses a template called “a photo of a [CLASS]” as a text prompt. A prompt can be formulated as:

(5) $$t_{j}=\left[\mathrm{Vector}\right]\left[\;{\mathrm{CLASS}}_{\;j}\right]$$

Theoretically, vectors can be arbitrary, because frozen, pre-trained VLMs that can quickly adapt provide new approaches. Adjusting the context of the text prompt can significantly affect the ability of the target dataset [13,35]. For a more detailed design, please refer to CLIP’s [1] original work.

#### 3.1.1. MDAPT Design

Our aim is to optimize the text prompt and visual prompt. Following Muhammad Uzair Khattak, Hanoona Rasheed, and Muhammad Maaz et al. [31,47], we designed deep MDAPT. We introduce *b* learnable tokens $\{P^{i}\in R^{d_{l}}{\}}_{i=1}^{b}$, in the language branch of CLIP. The input embeddings now follow the form $\left[P^{1},p^{2},\cdot \cdot \cdot ,P^{b},W_{0}\right]$, where $W_{0}=\left[\omega^{1},\omega^{2},\cdot \cdot \cdot ,\omega^{N}\right]$ corresponds to fixed input tokens. New learnable tokens are further introduced in each transformer block of the language encoder $L_{i}$ up to a specific depth *J*, and its formula is described as follows:

(6) $$\left[\_,W_{i}\right]=L_{i}\left(\left[P_{i-1},W_{i-1}\right]\right)\;i=1,2,\cdot \cdot \cdot \;,J.$$

Here, $\left[\cdot ,\cdot \right]$ refers to the concatenation operation.After the *J*th transfomer layer, subsequent layers process the previous layer prompts.

(7) $$\left[P_{j},W_{j}\right]=L_{j}\left(P_{j-1},W_{j-1}\right)\;\;j=J+1,2,\cdot \cdot \cdot \;,K$$

where *K* represents the maximum number of transformer encoding layers and $L_{i}$ represents the computation of a particular transfomer layer. When J=1, it represents the shallow MDAPT. Only the learnable parameters of the first layer are updated. Finally, the final text representation $z_{t}$ is computed.

(8) $$z_{t}=TextProj\left(w_{K}^{N}\right)$$

To ensure coordination between visual languages, vision prompts $\tilde{P}$ are obtained by projecting the textual prompts *P* onto the textual projection, which we call the visual language coupling function F(·). The formula is as follows:

(9) $${\tilde{P}}_{k}=F_{k}\left(P_{k}\right)$$

The coupling function is implemented as a linear layer that maps $d_{l}$-dimensional inputs to $d_{v}$-dimensions; $d_{l}$ and $d_{v}$ represent the text coding layer dimension and the visual coding layer dimension, respectively.

Similarly, we introduce b learnable tokens, $\{{\tilde{P}}^{i}\in R^{d_{l}}{\}}_{i=1}^{b}$, in the vision branch of CLIP alongside the input image tokens. New learnable tokens are further introduced in deeper transformer layers of the image encoder $V_{i}$ up to depth *J*. The deep visual prompts formula is expressed as follows:

(10) $$\left[c_{i},E_{i},\_\right]=V_{i}\left(\left[c_{i-1},E_{i-1},{\tilde{P}}_{i-1}\right]\right)\;i=1,2,\cdot \cdot \cdot \;,J.$$

(11) $$\left[c_{j},E_{j},{\tilde{P}}_{j}\right]=V_{j}\left(c_{j-1},E_{j-1},{\tilde{P}}_{j-1}\right)\;\;j=J+1,2,\cdot \cdot \cdot \;,K.$$

where *c* is the category embedding and *E* is the vision module embedding.

Finally, the image representation $z_{v}$ is computed.

(12) $$z_{v}=ImageProj\left(c_{K}\right)$$

#### 3.1.2. Project Gradient Descent (PGD)

Projected Gradient Descent (PGD) [12] is a very important optimization algorithm, which has a wide range of applications in machine learning, deep learning, computer vision, and other fields. PGD is an iterative algorithm mainly used to find the minimum value of the loss function. It minimizes the loss function by updating the parameters according to the direction of gradient descent in each iteration step. Compared with other optimization algorithms, PGD has many advantages, such as it is insensitive to the selection of initial points, it can handle constrained problems, and it is also very fast. Suppose we have a classification model f(·) with input *x*, label *y*, and loss function L(·,·), and we want the resulting adduced sample $x^{\prime }$ to maximize the loss function *L*. Here is the formula for a PGD attack:

1. Initialize the disturbance δ:
  (13) $$\delta_{0}=P\left(z\right);\;z∼U\left(-\epsilon ,\epsilon \right)$$
  where P is the operation that projects *z* onto the ϵ− neighborhood of *x*, and U(−ϵ,ϵ) represents a uniformly distributed random variable over the range [−ϵ,ϵ].
2. Iterative update perturbation δ: for i=1,2,…,k:
  (14) $$\delta_{i}=\delta_{i-1}+\alpha \cdot \mathrm{sign}\left(\nabla_{x}L\left(f\left(x+\delta_{i-1}\right),y\right)\right)$$
  where α is the step size of perturbation update, *k* is the number of update steps, and sign(·) is the direction sign of the update along the gradient direction.
3. The shadow operation ensures that $\delta_{i}$ is in the ϵ− neighborhood of *x*:
  (15) $$\delta_{i}=P\left(x+\delta_{i}-x\right)$$
  This is usually done by clipping each element of $\delta_{i}$:
  (16) $$\delta_{i}=\min\left(\max\left(\delta_{i},-\epsilon \right),\epsilon \right)$$
4. Final counter sample $x^{\prime }$:
  (17) $$x^{\prime }=x+\delta_{k}$$

#### 3.1.3. Parameter Optimization

To enhance adversarial robustness, we employ adversarial training [12] to train the prompt context. The formula is as follows:

(18) $$\arg\;\min_{t,\hat{t}}E_{i\in D}L\left(x_{i}+\delta_{i},t,{\hat{t}}_{i},y_{i};\theta_{v},\theta_{t}\right)$$

where $E_{i\in D}$ represents an expectation calculation for all samples in the entire training set D, with the goal of updating the model based on the loss of all samples. Disturbance $\delta_{i}$ is generated by the above PDG algorithm [12], and the essence of our optimization goal is to optimize the text prompt vector *t* and the visual prompt vector $\hat{t}$.

## 4. Experiments

In this section, we compare MDAPT with the state-of-the-art methods by using it on three datasets: Caltech101 [48],Flowers102 [49], and StanfordCars [50]. We also performed ablation experiments to assess the technical impact of MDAPT implementation.

### 4.1. Implementation Details

Our experiments are based on the following setup, unless otherwise stated (see Appendix A for a more detailed setup). Due to limited computational resources, we used fewer samples for training and evaluation: 50 epochs of 1-shot training, 100 epochs of 4-shot training, and 200 epochs of 16-shot training. Each method is evaluated on a testset. The backbone of the model image encoder we use is VIT-B/32 [10], and the weights of the image encoder are pre-trained by using the zero-shot adversarial robustness method TeCoA [51].

### 4.2. Datasets

Caltech101 [48] is a well-known computer vision dataset mainly used for image classification tasks. This dataset, proposed by the California Institute of Technology, contains a large number of images of different categories and is widely used to evaluate the performance of image classification algorithms. The Caltech101 dataset contains 101 different object categories. Each category contains a certain number of images, and the category types include animals, plants, everyday objects, etc. For example, the categories in the dataset are backgrounds, kites, electric guitars, whales, chickens, airplanes, and so on.

Flowers102 [49] is a commonly used dataset for image classification tasks, especially for plant classification and object recognition research. It was created by the Vision and Learning Laboratory at the University of Cambridge and has been widely used for model evaluation in the field of computer vision, especially for the application of deep-learning algorithms to plant classification tasks. The Flowers102 dataset contains 102 different flower categories, each corresponding to a flower species. It includes common flowers such as roses, chrysanthemums, sunflowers, tulips, and poppies.

StanfordCars [50] is a standard dataset widely used for image classification and object detection, specifically for cars. Released by Stanford University, the dataset is mainly used for research on car brand and model recognition and is widely used for various image classification and object recognition tasks in the fields of computer vision and deep learning. The StanfordCars dataset contains 196 car categories, each representing a different car model or brand. For example, it includes different types of luxury cars, sports cars, SUVs, trucks, sedans, etc., from several well-known brands such as Mercedes-Benz, BMW, Audi, Toyota, etc.

### 4.3. Evaluation Metrics

In this paper, we use accuracy and robustness to evaluate the performance of the model, and the accuracy and robustness formulas are shown below:

(19) $$Accuracy=\frac{TP+TN}{TP+TN+FP+FN}$$

where TP represents samples where the model predicts a positive class and the true label is also a positive class, TN represents samples where the model predicts a negative class and the true label is also a negative class, FP represents samples where the model incorrectly predicts a positive class but the true label is a negative class, and FN represents samples where the model incorrectly predicts a negative class but the true label is a positive class.

(20) $$Robustness=\frac{Number\;of\;Correct\;Predictions\;on\;Adversarial\;Examples}{Total\;Number\;of\;Adversarial\;Examples}$$

The metric of robustness is an important indicator of the model’s resistance to input perturbations. In the case of adversarial samples, one of its metrics responds to whether or not the model can still maintain a high ability to correctly classify.

### 4.4. Contrast Experiment

Our approach will be compared with four effective methods, namely Hand-Engineered Prompts (HEP) [1], Partial Adversarial Fine-Tuning PAFT [14], Adversarial Visual Programming (AVP) [17], and Adversarial Prompting Programming (APT) [19]. HEP was first proposed by CLIP, and then widely used [13]. We use “a photo of a” as a manual prompt. For the parameter adaptive approach, we compare it to PAFT and APT. We use PGD [12] for adversarial attacks. All compared methods share the same frozen pre-trained images and text encoder. Table 1 shows the comparison results of these methods.

**Figure 4:**
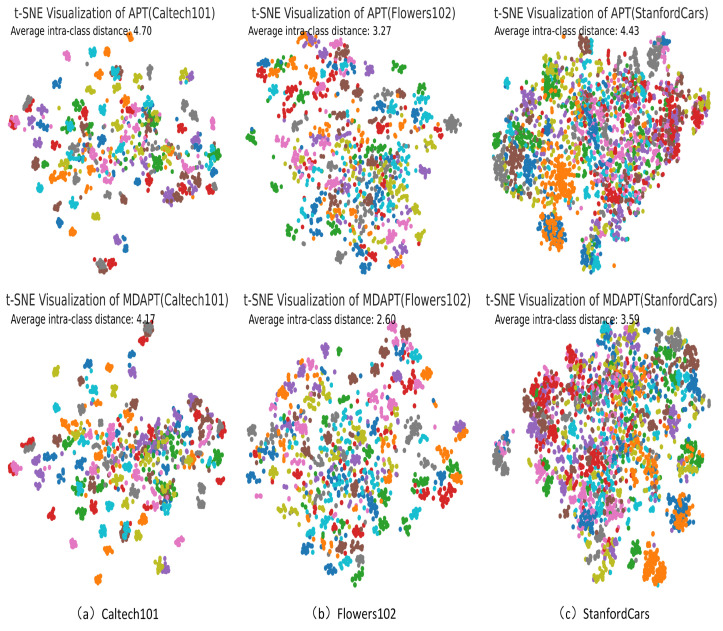
Using t-SNE [52] to visualize the image embedding distributions of the three datasets. The same colour represents the same category.Our method has a smaller average intra-class distance compared to unimodal APT; therefore, it can better cluster the same class.

Meanwhile, we use t-SNE [52] to describe the visualization of the image features embeddings distribution, as shown in Figure 4. Compared with unimodal APT, the average intra-class distance of our image feature embeddings distribution is smaller. Consequently, our method of aggregating the same class shows superior results, making it effective in strengthening adversarial robustness.

As can be seen in Table 1, our method has the best average accuracy and robustness. Compared to HEP, the average accuracy is improved by 17.84% and robustness is improved by 10.85%, and compared to PAFT, the average accuracy is improved by 9.84% and robustness is improved by 1.39%. Compared to the AVP algorithm, the algorithm improved the average accuracy by 17.54% and robustness by 10.75%. Compared to APT, the average accuracy is improved by 2.43% and the average robustness is improved by 2.42%, which improves the accuracy and robustness and achieves a better balance.

Comparing with HEP, our algorithm improves the accuracy by 17.84% and robustness by 10.85%, but HEP may be advantageous in dealing with some specific situations (e.g., computational complexity, etc.). Comparing with PAFT and APT, although our method has a large improvement, PAFT and APT may perform better under some specific tasks, and they may be easier to train and optimize.

#### 4.4.1. Adversarial Sample Visualization

**Figure 5:**
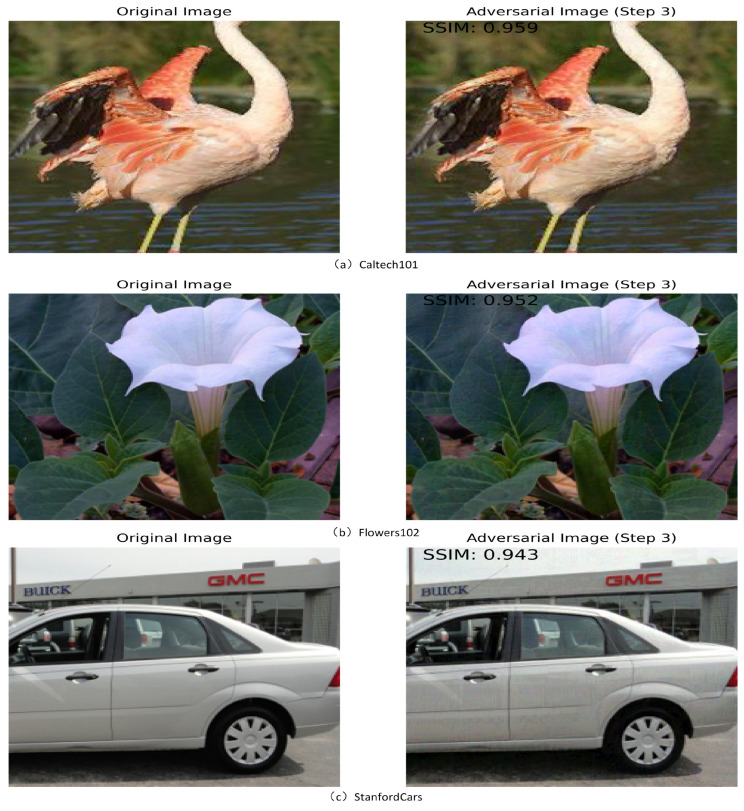
(**a**–**c**) Adversarial samples generated during adversarial training for the Caltech101, Flowers102, and StanfordCars datasets, respectively. SSIM [53] is a metric used to measure the structural similarity between two images. By the SSMI metrics, it can be seen that there are some perturbations in the adversarial image relative to the original image.

We use the PGD algorithm for adversarial training, with adversarial perturbations ϵ=4/255 and a step size of 3, which is used to limit the update step size α in the adversarial example generation process to 2.67. The adversarial samples generated during adversarial training are shown in Figure 5. SSMI [53] is a visual perception-based metric that evaluates the similarity of images in terms of brightness, contrast, and structure. The value of SSIM ranges from −1 to 1, where 1 means that the two images are exactly the same and −1 means that they are completely different. By the SSMI metrics, it can be seen that there are some perturbations in the adversarial image relative to the original image, which will help in multi-modal depth adversarial prompt tuning.

#### 4.4.2. Evaluation of Results on Caltech101 Dataset

Table 2 shows how Caltech101 compares to other methods. Our overall average performance outperforms the other methods. Compared with HEP, our accuracy is improved by 4.83% on average and robustness by 7.05% on average. The accuracy is improved by 0.12% on average and robustness by 1.09% on average compared to APT.

#### 4.4.3. Evaluation of Results on Flowers102 Dataset

Table 3 compares the performance of the different methods on the Flowers102 dataset. Compared to HEP, the average improvement in accuracy is 28.61% and the average improvement in robustness is 19.78%. Compared to APT, the average improvement in accuracy is 1.86% and the average improvement in robustness is 4.6%.

#### 4.4.4. Evaluation of Results on StanfordCars Dataset

Table 4 compares the performance of the different methods on the StanfordCars dataset. Compared to HEP, the average improvement in accuracy is 19.66% and the average improvement in robustness is 5.72%. Compared to APT, the average improvement in accuracy is 4.9% and the average improvement in robustness is 1.56%.

#### 4.4.5. Discussion of Performance Differences Between Datasets

The reason for the better performance on Caltech101 than on StanfordCars may be due to the difference in the nature of the two datasets; Caltech101 contains 101 categories, with large differences between the categories, which are easy for the model to differentiate between, whereas Stanford Cars concentrates on the automobile category, which has a high degree of similarity between different models, making it more difficult to categorize.

The Flowers102 dataset contains images of flowers, with relatively large visual differences between categories and simple backgrounds, which are easy for the model to distinguish. The StanfordCars dataset, on the other hand, contains 196 automobile categories, with high similarity in appearance between categories, and complex camera angles and backgrounds, which increase the difficulty of categorization, and thus the model’s performance on this dataset is weaker.

In addition, the size of the dataset and the number of categories also affect the model performance. The StanfordCars dataset is large and has many categories, which may make it more difficult to capture the features of all the categories during the training process. Relatively speaking, Flowers102 and Caltech101 both have fewer categories, making it easier to improve accuracy and robustness.

### 4.5. Ablation Experiment

#### 4.5.1. Prompt Length

To investigate the effect of prompt size on experimental results, we performed prompt ablation experiments. In the case of ϵ=4/255, a prompt depth of 1, and a shot of 16, we evaluated the effect of prompt length on accuracy and robustness. We evaluated the prompt lengths of 1, 4, 8, and 16 and averaged the results of the three datasets, as shown in Table 2. From Table 5, it can be seen that accuracy and robustness increase as the prompt length increases. Therefore, our default prompt size is 16.

#### 4.5.2. Prompt Depth

Prompt depth is also important, and we have explored related experiments. We evaluated the performance for different prompt depths, again, in the case of ϵ=4/255, prompt length of 16, and shot of 16; we evaluated the average results for the three datasets at prompt depths of 1, 3, 6, 9, and 12, as shown in Table 6. As can be seen from the table, the performance increases with the prompt depth, and the accuracy and robustness are highest at a depth of 9, and then the performance decreases. Therefore, we chose a prompt size of 16 and a prompt depth of 9 to evaluate our more in-depth approach.

#### 4.5.3. Impact of ϵ Size

The ϵ parameter is also very important as it represents the intensity of adversarial training. We explored the effect of different ϵ sizes on performance. With a prompt size of 16, a prompt depth of 9, and 16 shot of training 200 epochs, our average experimental results are shown in Table 7. From the table, it is quite reasonable that the results of the adversarial prompt tuning evaluation decrease as the ϵ parameter increases.

#### 4.5.4. Different Methods of Attack

For the generalization of the model attack, we evaluated the impact of different attack methods on the model performance. In the case of ϵ=4/255, prompt length of 16, shot of 16, and prompt depth of 9, we conducted attack experiments using the TPGD [54] and CW [55] attack methods. As shown in Table 8, our approach achieves the best performance for all three different algorithms. This also shows that our method is not specific to any particular attack or obfuscates the gradient overfitting result.

## 5. Conclusions

In this paper, we have studied the multi-modal approach of text-prompt guided generation to improve the robustness of pre-trained visual language models. We propose a novel method called Multi-modal Depth Adversarial Prompt Tuning (MDAPT) to enhance the robustness of CLIP against adversarial perturbations. Through extensive experiments on three datasets, we have demonstrated the effectiveness of MDAPT in improving performance and various attack methods.

## Figures and Tables

**Table 1:** The average performance of the three datasets under different shots. The HEP is manually adjusted on the target datasets, so there is no strict control over the number of shots used and all shots have the same result.

Method	1shot		4shot		16shot		Average	
	acc	rob	acc	rob	acc	rob	acc	rob
HEP [1]	39.63	18.08	39.63	18.08	39.63	18.08	39.63	18.08
PAFT [14]	28.37	16.94	47.87	25.95	66.66	39.73	47.63	27.54
AVP [17]	39.73	17.81	39.90	18.08	40.15	18.66	39.93	18.18
APT [19]	43.59	18.71	56.76	26.85	64.76	33.98	55.04	26.51
MDAPT(ours)	42.81	21.20	57.63	27.40	71.97	38.19	**57.47**↑	**28.93**↑

**Notes:** Bold numbers represents the best average performance of our method. The arrow (↑) indicates the improvement of this metric.

**Table 2:** Results of comparing our method with other validity methods on the Caltech101 dataset.

Method	1shot		4shot		16shot		Average	
	acc	rob	acc	rob	acc	rob	acc	rob
HEP	77.20	44.05	77.20	44.05	77.20	44.05	77.20	44.05
PAFT	51.48	33.51	73.02	45.55	86.12	60.40	70.21	46.48
AVP	77.40	43.12	77.44	43.57	77.40	44.18	77.41	43.62
APT	76.59	42.03	82.88	51.27	86.28	56.75	81.91	50.01
MDAPT(ours)	78.09	47.26	82.65	48.14	84.55	57.89	**82.03**↑	**51.10**↑

**Notes:** Bold numbers represents the best average performance of our method. The arrow (↑) indicates the improvement of this metric.

**Table 3:** Results of comparing our method with other validity methods on the Flowers102 dataset.

Method	1shot		4shot		16shot		Average	
	acc	rob	acc	rob	acc	rob	acc	rob
HEP	31.38	9.26	31.38	9.26	31.38	9.26	31.38	9.26
PAFT	28.05	15.14	56.43	28.54	79.54	47.87	54.67	30.51
AVP	31.42	9.38	31.59	9.58	31.55	10.27	32.52	9.74
APT	35.93	11.49	62.04	24.32	76.41	37.51	58.13	24.44
MDAPT(ours)	32.80	12.79	65.16	29.15	82.05	45.19	**59.99**↑	**29.04**↑

**Notes:** Bold numbers represents the best average performance of our method. The arrow (↑) indicates the improvement of this metric.

**Table 4:** Results of comparing our method with other validity methods on the StanfordCars dataset.

Method	1shot		4shot		16shot		Average	
	acc	rob	acc	rob	acc	rob	acc	rob
HEP	10.32	0.92	10.32	0.92	10.32	0.92	10.32	0.92
PAFT	5.58	2.18	14.18	3.76	34.32	10.92	18.03	5.62
AVP	10.38	0.95	10.67	1.08	11.50	1.52	10.85	1.18
APT	18.26	2.61	25.37	4.94	31.60	7.70	25.08	5.08
MDAPT(ours)	17.54	3.54	25.08	4.91	49.32	11.48	**29.98**↑	**6.64**↑

**Notes:** Bold numbers represents the best average performance of our method. The arrow (↑) indicates the improvement of this metric.

**Table 5:** Our method averages performance with a different number of context vectors, where *N* stands for prompt size.

*N*	Accuracy	Robustness
1	58.68	28.42
4	61.43	30.79
8	63.31	31.98
16	**65.06**	**33.76**

**Notes:** Bold numbers represent the best performance.

**Table 6:** Our method averages the performance for different prompt depths, where *J* stands for prompt depth.

*J*	Accuracy	Robustness
1	65.06	33.76
3	67.67	35.49
6	69.89	36.81
9	**71.97**	**38.19**
12	69.25	36.42

**Notes:** Bold numbers represent the best performance.

**Table 7:** The performance of our method for different ϵ sizes; ϵ represents the adversarial training intensity size.

ϵ	Accuracy	Robustness
1/255	84.29	69.86
4/255	71.97	38.19

**Table 8:** Compared with other methods, our approach is highly effective in three different attack methods on three datasets. Our adversarial assessment is highly reliable, while also achieving a balance between accuracy and robustness.

Method	Accuracy	PGD	TPGD	CW	Average
HEP	39.63	18.08	30.10	17.52	21.90
APT	64.76	33.97	37.37	23.88	31.74
MDAPT(ours)	**71.79**↑	**38.19**↑	**53.35**↑	**37.17**↑	**42.90**↑

**Notes:** Bold numbers represents the best average performance of our method. The arrow (↑) indicates the improvement of this metric.

## Data Availability

The data presented in this study are openly available.

## Appendix A

The MDAPT training environment is Ubuntu version 22.04, GPU 4070ti, pytorch1.12.0. Training was carried out using Stochastic Gradient Descent (SGD) with cosine learning rate scheduling with an initial learning rate of 0.002. Also, random initialization, size of X prompt sizes, and a batch size of 128 are used. For all datasets, 1-shot training for 50 epochs, 4-shot training for 100 epochs, and 16-shot training for 200 epochs are performed. Our experimental attack algorithm for adversarial training and evaluation is PGD [12]. Our experimental setup for adversarial training and evaluation is PGD, which is used for both training and evaluation, with adversarial perturbations ϵ=4/255 and a step size of 3, which is used to limit the update step size α in the adversarial example generation process to 2.67. As described in the literature [19], the inference prompt is used for the attack with the highest attack strength; therefore, we set the inference prompts equal to the attack prompts. For estimation, we use testsets for evaluation. APT was trained by Stochastic Gradient Descent (SGD) using cosine learning rate scheduling with an initial learning rate of 0.002. The batch size was 32. The APT was trained 1 shot for 50 epochs, 4 shot for 100 epochs and 16 shot for 200 epochs. The AVP was designed with a prompt size of 52; we used a cosine learning rate with an initial learning rate of 0.1; and we set the epochs for the 1-shot, 4-shot, and 16-shot trainings to be 20, 50, and 100 epochs, respectively. In accordance with Tianlong Chen, Sijia Liu, and Shiyu et al. [14], for the PAFT training, 1 shot, 4 shot, and 16 shot were trained for 20, 50, and 100 epochs, respectively, with an initial learning rate of 0.1, decaying by 0.1 at 5, 15, and 30 epochs and 10, 25, and 50 epochs.
